# BMI trajectory of rapid and excessive weight gain during adulthood is associated with bone loss: a cross-sectional study from NHANES 2005–2018

**DOI:** 10.1186/s12967-023-04397-9

**Published:** 2023-08-12

**Authors:** Jiacheng Wang, Yi Zheng, Yawen Wang, Chengjun Zhang, Yanfeng Jiang, Chen Suo, Mei Cui, Tiejun Zhang, Xingdong Chen, Kelin Xu

**Affiliations:** 1 School of Public Health, and the Key Laboratory of Public Health Safety of Ministry of Education, Fudan University, Shanghai, 200000 China; 2 State Key Laboratory of Genetic Engineering, Human Phenome Institute, Fudan University, Shanghai, 200000 China; 3 Fudan University Taizhou Institute of Health Sciences, Taizhou, Jiangsu China; 4 National Clinical Research Center for Aging and Medicine, Huashan Hospital, Fudan University, Shanghai, China; 5 Department of Neurology, Huashan Hospital, Fudan University, Shanghai, China; 6 Yiwu Research Institute of Fudan University, Yiwu, Zhejiang China

**Keywords:** Body mass index, Weight change, Osteoporosis, Bone mineral density, NHANES

## Abstract

**Background:**

Studies have examined the effect of weight change on osteoporosis, but the results were controversial. Among them, few had looked at weight change over the life span. This study aimed to fill this gap and investigate the association between lifetime body mass index (BMI) trajectories and bone loss.

**Methods:**

In this cross-sectional study, participants at age 50 and above were selected from the National Health and Nutrition Examination Survey (NHANES) 2005–2018. Dual-energy X-ray Absorptiometry was used to measure the bone mineral density at the femoral neck and lumbar spine. Standard BMI criteria were used, with < 25 kg/m^2^ for normal, 25–29.9 kg/m^2^ for overweight, and ≥ 30 kg/m^2^ for obesity. The latent class trajectory model (LCTM) was used to identify BMI trajectories. Multinomial logistic regression models were fitted to evaluate the association between different BMI trajectories and osteoporosis or osteopenia.

**Results:**

For the 9,706 eligible participants, we identified four BMI trajectories, including stable (n = 7,681, 70.14%), slight increase (n = 1253, 12.91%), increase to decrease (n = 195, 2.01%), and rapid increase (n = 577, 5.94%). Compared with individuals in the stable trajectory, individuals in the rapid increase trajectory had higher odds of osteoporosis (OR = 2.25, 95% CI 1.19–4.23) and osteopenia (OR = 1.49, 95% CI 1.02–2.17). This association was only found in the lumbar spine (OR = 2.11, 95% CI 1.06–4.2) but not in the femoral neck. In early-stage (age 25–10 years ago) weight change, staying an obesity and stable weight seemed to have protective effects on osteoporosis (OR = 0.26, 95% CI 0.08–0.77) and osteopenia (OR = 0.46, 95% CI 0.25–0.84). Meanwhile, keeping an early-stage stable and overweight was related to lower odds of osteopenia (OR = 0.53, 95% CI 0.34–0.83). No statistically significant association between recent (10 years ago to baseline) weight change and osteoporosis was found.

**Conclusions:**

Rapid and excess weight gain during adulthood is associated with a higher risk of osteoporosis. But this association varies by skeletal sites. Maintaining stable overweight and obesity at an early stage may have potentially beneficial effects on bone health.

**Supplementary Information:**

The online version contains supplementary material available at 10.1186/s12967-023-04397-9.

## Background

Osteoporosis is a systemic skeletal metabolic ailment characterized by decreased bone mass and disturbance of bone architecture, which compromises bone strength and raises the risk of fracture [1]. According to a meta-analysis, the global prevalence of osteoporosis and osteopenia were estimated to be 19.7% and 40.4%, respectively [2]. The National Health and Nutrition Examination Survey (NHANES) data report from 2005 to 2010 showed that 48.3% of individuals aged 65 years and over had osteopenia at the lumbar spine or femoral neck, and 16.2% of the persons had osteoporosis in the United States [3]. However, as the population ages, the prevalence of osteoporosis is expected to increase even further [4].

Body mass index (BMI) and bone mineral density (BMD) are only significantly correlated at certain ages [5, 6]. Yet, studies on the association between BMI and osteoporosis have produced inconsistent results [7, 8], possibly because most studies utilized BMI at a single time point, which ignored the influence of longitudinal weight fluctuation on osteoporosis. Recent studies have focused on the relationship between weight changes and osteoporosis. K E Ensrud et al. demonstrated that late-life weight loss in males was associated with lower total BMD and weaker peripheral bones [9]. A cross-sectional study revealed that the increased likelihood of osteoporosis was significantly affected by attempting to gain weight or loss weight [10]. Nevertheless, most of the research assessed BMI at two time points and did not consider weight changes over a lifetime. Such an approach neglects the course of BMI trajectories over the lifespan, which, given the varying trends, rates, and magnitudes of BMI change, might provide further insight into the complex association of weight change with bone loss.

Based on BMI data from multiple time points, this study used the latent class trajectory model (LCTM) to identify BMI change trajectories during participants’ lifespans. And we studied the association between different BMI trajectories and osteoporosis or osteopenia using multinomial logistic regression among people aged 50 and older from NHANES 2005–2018. From the perspective of weight change trajectories, our present study may provide some strategies for the prevention of osteoporosis in the middle-aged and elderly population.

## Methods

### Study population and participants selected

The NHANES is a series of cross-sectional national surveys conducted by the National Center for Health Statistics (NCHS) to assess and study the health and nutrition status of American adults and children. Stratified multi-stage sampling techniques and documented designs are used to ensure the sample is representative of the civilian American population [11]. The ethics and data collection protocols were approved by the NCHS Ethics Review Board, and all participants signed informed consent before the interviews and health examinations. The data used in this study were acquired from the NHANES website at https://www.cdc.gov/nchs/nhanes/index.htm.

**Fig. 1:**
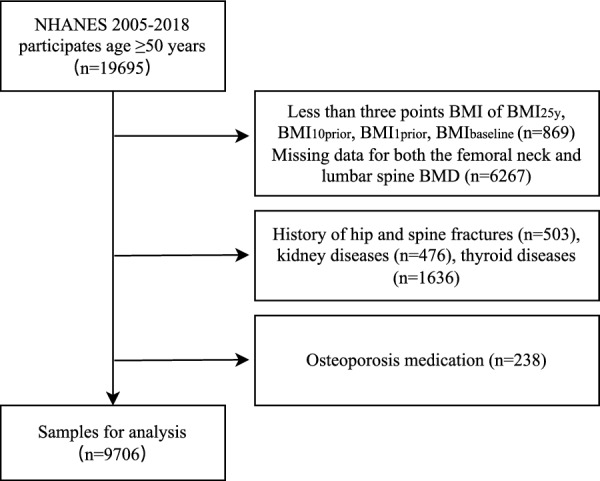
Flow chart of inclusion and exclusion criteria from the NHANES 2005–2018 database

This study was a cross-sectional study and involved a secondary analysis. Data sets were selected from NHANES 2005–2018. Participants aged 50 and above were enrolled, similar to other studies [12, 13]. We excluded the participants with less than three BMI records and have missing data for both the femoral neck and lumbar spine BMD. We also excluded those with a history of hip and spine fractures, kidney diseases, thyroid disease, and osteoporosis medication to ensure that the results were not confounded by these factors. Finally, a total of 9,706 participants were included in the study analyses (Fig. 1). In the following, we referred to the “baseline” as the time when the survey was conducted and all covariates were collected.

### Definition of BMI and weight change

The NHANES Anthropometry section measured participants’ standing height and weight at baseline using standardized examination procedures [14]. The Sample Person Questionnaire retrospectively collected self-reported weight history, including weight one year ago, weight 10 years ago, and weight and height at age 25. We estimated the height 1 year ago and 10 years ago by using the baseline height. BMIs (BMI25y, BMI10prior, BMI1prior, and BMIbaseline) were calculated as weight (kg) at the specific time divided by the corresponding height (m) squared, respectively. For example, BMI25y was defined as weight at age 25 divided by height squared at age 25. BMI data more than three times the interquartile range (IQR) were treated as missing values in our study.

To investigate weight change patterns across different life stages, we evaluated weight changes during the early stage (from 25 years old to 10 years ago) and the recent stage (from 10 years ago to baseline). We considered a weight change of 5% or less as stable and a change of over 5% as significant [15, 16]. Standard BMI criteria were used, with < 25 kg/m^2^ for normal, 25–29.9 kg/m^2^ for overweight, and ≥ 30 kg/m^2^ for obesity. We defined weight change patterns by considering BMI status at the previous time point and the weight change percentage between the two time points. For instance, “normal-stable” in the early stage was defined as having a normal BMI at age 25 and no more than a 5% weight change from age 25 to 10 years ago. The same criterion was applied to define “overweight-stable” and “obesity-stable” weight change patterns. Participants whose BMI changed by more than 5% were assigned to either the “increase” or “decrease” group.

### BMD measurement and definition of osteoporosis/osteopenia

Dual-energy X-ray Absorptiometry (DXA) is widely used as the gold standard of bone density assessment due to its validation and the low dose of X-rays [17]. The DXA examination protocol is well-documented in the Body Composition Procedures Manual located on the NHANES website. DXA scans were collected for both the femoral neck and lumbar spine in NHANES 2005–2010, 2013–2014, and 2017–2018. The Lumbar spine was scanned in NHANES 2011–2012 and 2015–2016. The bone mineral density T-score was calculated using the formula (BMD_respondent_-mean BMD_reference group_)/SD_reference group_. As recommended by the World Health Organization (WHO) and the International Society for Clinical Densitometry (ISCD) [18], we used non-Hispanic white females aged 20–29 years from NHANES III data as the reference group for femoral neck measurements [19], while the reference group for lumbar spine measurements was obtained from the Vital and Health Statistics released by the Centers for Disease Control and Prevention (CDC) [20]. Osteoporosis was defined as a T-score ≤ −2.5 and osteopenia −2.5 < T-score ≤ −1.

### Covariates

Various sociodemographic information was obtained, including age, sex, ethnicity, education, marital status, and poverty-income ratio (PIR). PIR was calculated by dividing family income by poverty guideline, considering the family size, year, and state. A higher PIR reflects a relatively higher socioeconomic status [21]. We categorized PIR as ≤ 1.30, 1.31–3.50, and > 3.50 [22]. Smoking cigarettes was categorized as never, former, and current. Alcohol consumption was classified as non-drinker and ever-drinker, and ever-drinker was further divided into current drinker and social drinker based on drinking frequency [23]. Physical activity was categorized into inactive, insufficient, moderate, and high by the cut-off values of 600 and 1200 metabolic equivalents of task (MET) minutes per week in conformity with the Global Physical Activity Questionnaire Analysis [24]. We categorized sleep status into ≥ 7 h and < 7 h as suggested by the National Sleep Foundation [25]. Other covariates included cancer, diabetes, systolic/diastolic blood pressure (BP), total cholesterol, and baseline BMI. Cancer and diabetes were categorized into “yes” and “no” based on the questions “Ever told you had cancer or malignancy” and “Doctor told you to have diabetes”. BP (mmHg), total cholesterol (mmol/L), and baseline BMI (Kg/m^2^) were defined as continuous variables. In the NHANES study, BP was measured after the participants had been seated for five minutes, and three consecutive readings were obtained. A fourth BP reading was taken if the data was interrupted or incomplete. BP values were averaged across all measurements in our study.

### Statistical analysis

We used LCTM to identify the trajectories of BMI over time [26, 27]. The LCTM is a specialized type of finite mixture modeling that aims to find latent classes of people that exhibit similar trends in a determinant with time [28]. The optimal number of trajectories was chosen based on the minimum Bayesian information criteria (BIC) while maintaining the mean posterior probability over 70% in each class and the class size ≥ 2% of the population [29]. The model selection process also considered the clinical significance of the trajectories [30]. We assumed different BMI trajectories for men and women, due to sex differences in the factors that influence BMI [31]. Therefore, the LCTMs were fitted for men and women separately and used for subgroup analyses subsequently.

Baseline characteristic data were grouped by BMI trajectories and presented as weighted mean and standard error (SE) for continuous variables, and frequencies and weighted percentages for categorical variables. Analysis of variance (ANOVA) and chi-square tests were used for group comparisons of continuous and categorical variables, respectively. T-score comparisons of the femoral neck and lumbar spine across different BMI trajectories were presented using violin plots. Age and sex-adjusted partial correlation coefficients between the T-score and baseline BMI of the two skeletal sites were also calculated. We fitted three multinomial logistic regression models to evaluate the impact of different BMI trajectories on osteoporosis or osteopenia. To rule out the positive correlations between baseline BMI and BMD, baseline BMI was adjusted in all three models: Model 1: adjusted for age, sex, ethnicity, and baseline BMI; Model 2: Model 1 plus education, smoking, alcohol drinking, physical activity, and sleep status; Model 3: Model 2 plus diabetes, cancer, and total cholesterol. Results were reported as odds ratios (OR), 95% confidence intervals (95% CI), and *P*-values.

In sensitivity analyses, we excluded participants with extreme BMI values (< 15 and > 50 kg/m^2^) at any time point, as these values were deemed biologically implausible [26]. Furthermore, we calculated the E-value to gauge the potential influence of unobserved confounding on the observed association. To account for the complex sampling techniques and study design, all analyses used a weighted approach [32]. Statistical significance was indicated by a bidirectional *P*-value < 0.05. Data analyses were conducted using R software Version 4.2.1.

## Results

### The BMI trajectories and characteristics of participants

**Fig. 2:**
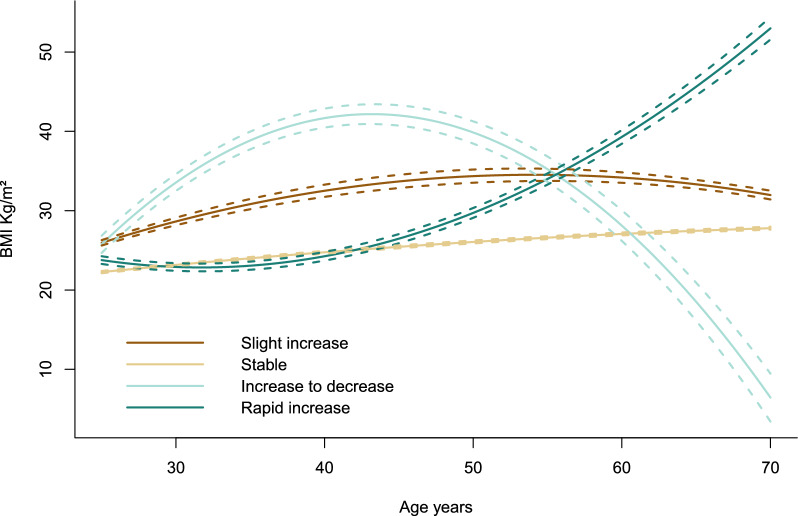
BMI trajectories from adulthood of the total population in NHANES 2005–2018. BMI trajectories were detected using the latent class trajectory model. The solid lines are the predicted BMI trajectory, and the dashed lines are the 95% confidence interval; *BMI* body mass index

Four BMI trajectories were identified using LCTM (Fig. 2). The values of the BIC parameter, the mean posterior probability, and the sample size of each trajectory in the model, which we used to determine the number of clusters, were presented in Additional file 1: Table S1. The majority of the participants remained on a stable trajectory during their lifetime (79.14%), while some experienced a slight increase in BMI (12.91%). 5.94% followed a moderately to rapidly increasing BMI trajectory throughout the time, and 2.01% had a trajectory of increasing to decreasing. These four trajectories were labeled as “stable,” “slight increase,” “rapid increase,” and “increase to decrease,” respectively. LCTM results for different sexes were presented in Additional file 1: Tables S2–S3 and Figure S1.

**Fig. 3:**
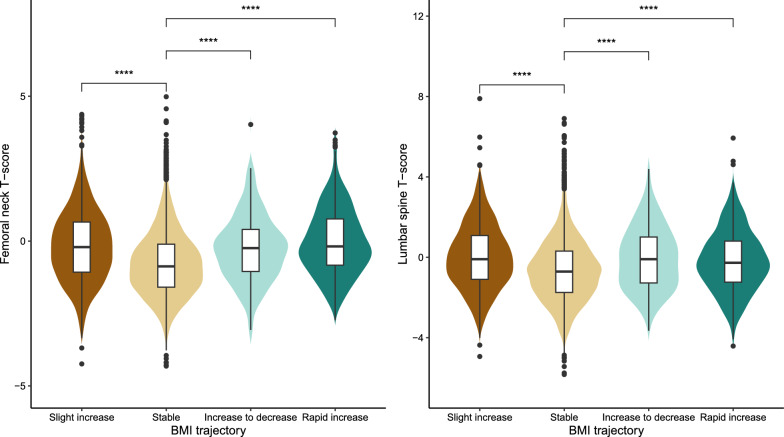
Violin plot for T-score comparisons of the femoral neck and lumbar spine across BMI trajectories; ****P < 0.05/3

Among all participants, the weighted mean age was 60.99 (0.16) years and 4,230 (45.24%) were female. The characteristics of the population were compared by trajectory groups in Table 1. Significant differences were observed in all variables except for education, diastolic blood pressure (DBP), and total cholesterol among the four groups. The stable trajectory group had the highest prevalence of osteoporosis and osteopenia but the lowest baseline BMI. And this group also exhibited the lowest T-scores for the femoral neck and lumbar spine (Fig. 3). We further calculated the partial correlation coefficients adjusted for age and sex between the T-score and baseline BMI of the two skeletal sites, resulting in partial correlation coefficients of 0.39 and 0.31 (both *P* < 0.001), respectively.

**Table 1:** Total population characteristics by BMI trajectories in NHANES 2005–2018

	Slight increase (n = 1252)	Stable (n = 7681)	Increase to decrease (n = 195)	Rapid increase (n = 577)	*P*-value
Age, mean, years	59.79 (0.30)	61.58(0.18)	56.05(0.55)	57.23(0.28)	** < 0.001**
Sex					** < 0.001**
Male	690 (58.26)	4498 (55.97)	82 (41.90)	206 (34.71)	
Female	563 (41.74)	3183 (44.03)	113 (58.10)	371 (65.29)	
Ethnicity					** < 0.001**
Mexican American	233 (7.89)	991 (4.78)	51 (12.16)	85 (6.48)	
Other Hispanic	108 (3.66)	694 (4.15)	18 (4.81)	56 (4.41)	
Non-hispanic white	495 (70.79)	3608 (75.01)	69 (66.66)	195 (65.00)	
Non-hispanic black	378 (14.53)	1554 (8.99)	49 (13.56)	219 (20.33)	
Other race	39 (3.13)	834 (7.08)	8 (2.82)	22 (3.79)	
Education					0.105
Less than high school	389 (18.47)	2009 (15.86)	65 (20.19)	168 (16.41)	
High school including GED	305 (28.25)	1867 (25.00)	58 (30.06)	151 (28.57)	
Some college or above	559 (53.28)	3805 (59.14)	72 (49.75)	258 (55.01)	
Marital status					**0.009**
Currently married	760 (66.77)	4927 (68.97)	109 (67.59)	316 (61.36)	
Formerly married	389 (24.79)	2276 (25.45)	66 (25.54)	202 (30.94)	
Never married	104 (8.44)	478 (5.57)	20 (6.86)	59 (7.70)	
Poverty-income ratio					**0.008**
≤ 1.30	328 (17.27)	1784 (15.06)	70 (27.43)	172 (20.30)	
1.31–3.50	450 (34.23)	2729 (34.54)	60 (31.87)	181 (32.97)	
> 3.50	362 (48.50)	2480 (50.40)	41 (40.69)	170 (46.73)	
Smoke					**0.016**
Non-smoker	668 (55.34)	3711 (49.04)	83 (46.63)	268 (48.63)	
Former smoker	399 (31.27)	2553 (49.04)	55 (26.57)	192 (33.61)	
Current smoker	185 (13.39)	1415 (17.50)	57 (26.80)	117 (17.75)	
Alcohol					** < 0.001**
Non-drinker	413 (29.82)	2115 (22.95)	64 (26.69)	194 (29.88)	
Current drinker	342 (33.47)	2723 (44.00)	52 (32.55)	141 (34.48)	
Social drinker	448 (36.70)	2519 (33.05)	67 (40.77)	212 (35.64)	
Physical activity					** < 0.001**
Inactive	424 (29.42)	2132 (23.00)	71 (38.54)	220 (34.65)	
Insufficient	242 (19.45)	1547 (19.56)	36 (14.22)	93 (16.08)	
Moderate	135 (11.41)	957 (12.65)	17 (7.78)	57 (12.94)	
High	452 (39.72)	3045 (44.79)	71 (39.47)	207 (36.33)	
Sleep status					** < 0.001**
≥ 7 h	759 (64.04)	5080 (69.25)	113 (67.32)	314 (56.74)	
< 7 h	493 (35.96)	2590 (30.75)	80 (32.68)	260 (43.26)	
Cancer					**0.024**
Yes	132 (12.41)	1120 (16.06)	22 (14.20)	59 (12.61)	
No	1121 (87.59)	6561 (83.94)	173 (85.80)	518 (87.39)	
Diabetes					** < 0.001**
Yes	415 (27.96)	1094 (10.11)	91 (45.72)	146 (23.36)	
No	838 (72.04)	6587 (89.89)	104 (54.28)	431 (76.64)	
SBP, mean, mmHg	128.50 (0.75)	128.31 (0.32)	126.15 (1.79)	131.98 (1.17)	**0.024**
DBP, mean, mmHg	72.30 (0.50)	71.68 (0.25)	71.19 (1.37)	72.45 (0.81)	0.999
Total cholesterol, mean, mmol/L	4.95 (0.05)	5.25 (0.02)	4.89 (0.09)	5.14 (0.06)	0.059
Current BMI	34.91 (0.20)	27.11 (0.07)	33.50 (0.97)	38.16 (0.30)	** < 0.001**
Bone health					** < 0.001**
Osteoporosis	64 (3.32)	814 (9.65)	8 (3.89)	30 (4.57)	
Osteopenia	331 (26.16)	3125 (40.17)	61 (26.81)	154 (27.82)	
Normal	858 (70.52)	3742 (50.19)	126 (69.3)	393 (67.61)	

BMI trajectories were identified by the latent class trajectory model, whose number of clusters was determined by the Bayesian information criteria, mean posterior probability, and sample sizes. Four BMI trajectories were generated, and labeled as “slight increase”, “stable”, “increase to decrease”, and “rapid increase” based on the shape of the trajectory chart (Fig. 2). Baseline characteristics were presented as the weighted mean and standard error (SE) for continuous variables; and the frequencies and weighted percentages for categorical variables

*SBP* systolic blood pressure, *DBP* diastolic blood pressure, *BMI* body mass index

*P*-values < 0.05 were indicated in bold

### Association between BMI trajectories and osteoporosis/osteopenia

We used multinomial logistic regression to assess the association between BMI trajectories and osteoporosis/osteopenia. As shown in Table 2, after adjustment for covariates, individuals in the rapid increase trajectory had 225% odds of developing osteoporosis (OR = 2.25, 95% CI 1.19–4.23, *P* = 0.012) and 149% odds of developing osteopenia (OR = 1.49, 95% CI 1.02–2.17, *P* = 0.040) compared with individuals in the stable trajectory. However, we found no statistically significant association between either a slight increase or increase to decrease trajectory and osteoporosis or osteopenia when compared to the stable trajectory. Sex-stratified analysis was consistent with that in the total population, where the odds of osteoporosis in the rapid increase trajectory were 2.69 (OR = 2.69, 95% CI 1.03–7.07, *P* = 0.044) and 2.62 (OR = 2.62, 95% CI 1.17–5.86, *P* = 0.019) times higher than the odds in the stable trajectory, for male and female, respectively. Participants were also stratified by age, and the statistically significant association between rapid increase trajectory and osteoporosis or osteopenia was only observed in the group ≤ 65 years. The odds ratios were 2.32 (95% CI 1.11–4.83, *P* = 0.025) for osteoporosis and 1.57 (95% CI 1.02–2.43, *P* = 0.043) for osteopenia.

**Table 2:** Multinomial logistic regression evaluates the association between BMI trajectories and osteoporosis/osteopenia

Stable	Osteoporosis						Osteopenia					
	Model 1	*P*-value	Model 2	*P*-value	Model 3	*P*-value	Model 1	*P*-value	Model 2	*P*-value	Model 3	*P*-value
	Ref.		Ref.		Ref.		Ref.		Ref.		Ref.	
Total population												
Slight increase	0.95 (0.67,1.33)	0.761	0.83 (0.58,1.18)	0.297	0.95 (0.66,1.37)	0.783	1.01 (0.82,1.25)	0.906	1.01 (0.82,1.24)	0.910	1.12 (0.89,1.40)	0.331
Increase to decrease	0.65 (0.27,1.54)	0.326	0.50 (0.20,1.27)	0.146	0.63 (0.26,1.58)	0.328	0.79 (0.46,1.34)	0.381	0.77 (0.45,1.34)	0.356	0.80 (0.51,1.27)	0.345
Rapid increase	2.32 (1.27,4.23)	**0.006**	2.06 (1.10,3.84)	**0.023**	2.25 (1.19,4.23)	**0.012**	1.35 (0.95,1.93)	0.095	1.38 (0.95,2.00)	0.090	1.49 (1.02,2.17)	**0.040**
Male												
Slight increase	1.79 (1.01,3.18)	**0.047**	1.66 (0.90,3.06)	0.106	1.87 (0.99,3.52)	0.052	1.03 (0.80,1.34)	0.802	1.08 (0.83,1.41)	0.573	1.16 (0.88,1.52)	0.285
Increase to decrease	1.47 (0.28,7.68)	0.646	1.04 (0.15,7.37)	0.972	1.56 (0.20,11.91)	0.671	0.69 (0.39,1.22)	0.200	0.66 (0.36,1.21)	0.180	0.82 (0.46,1.47)	0.504
Rapid increase	2.97 (1.15,7.64)	**0.024**	2.49 (0.98,6.38)	0.056	2.69 (1.03,7.07)	**0.044**	2.25 (1.27,4.00)	**0.006**	2.22 (1.19,4.14)	**0.012**	2.48 (1.31,4.69)	**0.005**
Female^§^												
Slight increase	0.80 (0.54,1.18)	0.262	0.73 (0.48,1.10)	0.129	0.82 (0.54,1.25)	0.352	0.98 (0.72,1.33)	0.896	0.92 (0.68,1.24)	0.577	1.02 (0.75,1.38)	0.909
Increase to decrease	0.64 (0.25,1.64)	0.355	0.59 (0.22,1.54)	0.280	0.64 (0.25,1.67)	0.363	0.82 (0.39,1.73)	0.604	0.85 (0.39,1.85)	0.687	0.74 (0.37,1.47)	0.391
Rapid increase	2.51 (1.19,5.29)	**0.016**	2.42 (1.09,5.39)	0.031	2.62 (1.17,5.86)	**0.019**	1.18 (0.76,1.83)	0.458	1.19 (0.77,1.85)	0.428	1.28 (0.83,1.97)	0.264
≤ 65 years												
Slight increase	0.84 (0.51,1.37)	0.481	0.73 (0.43,1.24)	0.243	0.88 (0.50,1.54)	0.643	1.01 (0.76,1.36)	0.935	1.03 (0.77,1.39)	0.839	1.15 (0.85,1.56)	0.371
Increase to decrease	0.53 (0.19,1.47)	0.223	0.38 (0.12,1.18)	0.093	0.52 (0.17,1.61)	0.257	0.76 (0.42,1.38)	0.370	0.75 (0.40,1.38)	0.353	0.77 (0.45,1.32)	0.337
Rapid increase	2.28 (1.13,4.58)	**0.021**	2.02 (0.99,4.14)	0.055	2.32 (1.11,4.83)	**0.025**	1.38 (0.92,2.06)	0.117	1.42 (0.93,2.17)	0.107	1.57 (1.02,2.43)	**0.043**
> 65 years												
Slight increase	1.04 (0.58,1.87)	0.883	0.92 (0.52,1.64)	0.779	1.00 (0.55,1.81)	0.990	1.03 (0.71,1.49)	0.893	0.98 (0.68,1.42)	0.924	1.07 (0.72,1.60)	0.741
Increase to decrease	1.39 (0.19,10.13)	0.746	1.64 (0.16,16.5)	0.672	1.80 (0.2,16.28)	0.603	1.08 (0.26,4.50)	0.916	1.44 (0.30,6.96)	0.647	1.55 (0.34,7.06)	0.570
Rapid increase	0.93 (0.21,4.10)	0.919	0.73 (0.15,3.57)	0.699	0.75 (0.15,3.81)	0.725	1.20 (0.57,2.50)	0.633	1.19 (0.56,2.52)	0.654	1.20 (0.56,2.60)	0.639

Model 1: adjusted for age, sex, ethnicity, and baseline BMI

Model 2: model 1 plus education, smoking, alcohol drinking, physical activity, and sleep status

Model 3: model 2 plus cancer, diabetes, and total cholesterol

^**§**^Additionally adjusted for menopause status in model 3

*P*-values < 0.05 were indicated in bold

### Different skeletal locations have different associations

Multinomial logistic regression analyses were also performed and adjusted for the same covariates in different skeletal sites. The results in Table 3 indicated that the association between BMI trajectory and osteoporosis varied depending on the bone site. In the femoral neck, we can only find a modest and positive association between rapid increase trajectory and osteopenia (OR = 1.45, 95% CI 1–2.09, *P* = 0.048) in model 3. As for the lumbar spine, the odds of osteoporosis were much higher in the rapid increase trajectory compared to the stable trajectory, across all models.

**Table 3:** The association between BMI trajectories and osteoporosis/osteopenia of different skeletal sites

Stable	Osteoporosis						Osteopenia					
	Model 1	*P*-value	Model 2	*P*-value	Model 3	*P*-value	Model 1	*P*-value	Model 2	*P*-value	Model 3	*P*-value
	Ref.		Ref.		Ref.		Ref.		Ref.		Ref.	
Femur neck												
Slight increase	1.21 (0.70,2.08)	0.497	1.06 (0.61,1.85)	0.839	1.13 (0.66,1.94)	0.645	1.11 (0.89,1.38)	0.346	1.10 (0.89,1.36)	0.393	1.21 (0.95,1.52)	0.116
Increase to decrease	1.02 (0.28,3.73)	0.975	0.85 (0.23,3.13)	0.812	0.99 (0.27,3.58)	0.985	0.77 (0.44,1.33)	0.345	0.73 (0.41,1.30)	0.279	0.72 (0.44,1.15)	0.169
Rapid increase	1.09 (0.15,7.89)	0.933	0.83 (0.11,6.42)	0.862	0.90 (0.12,6.99)	0.922	1.32 (0.93,1.88)	0.126	1.35 (0.94,1.94)	0.105	1.45 (1.00,2.09)	**0.048**
Lumbar spine												
Slight increase	0.90 (0.59,1.37)	0.615	0.78 (0.49,1.23)	0.282	0.90 (0.56,1.45)	0.660	0.96 (0.71,1.30)	0.811	0.93 (0.69,1.25)	0.632	1.00 (0.73,1.38)	0.978
Increase to decrease	0.73 (0.27,1.95)	0.531	0.57 (0.19,1.67)	0.305	0.74 (0.25,2.19)	0.593	1.00 (0.55,1.80)	0.988	0.99 (0.53,1.86)	0.984	0.96 (0.59,1.57)	0.881
Rapid increase	2.16 (1.13,4.10)	**0.019**	1.97 (1.00,3.88)	**0.049**	2.11 (1.06,4.20)	**0.034**	1.50 (0.99,2.28)	0.056	1.52 (0.98,2.36)	0.063	1.66 (1.05,2.63)	**0.029**

Model 1: adjusted for age, sex, ethnicity, and baseline BMI

Model 2: model 1 plus education, smoking, alcohol drinking, physical activity, and sleep status

Model 3: model 2 plus cancer, diabetes, and total cholesterol

*P*-values < 0.05 were indicated in bold

### Effect of weight change on osteoporosis/osteopenia at different life stages

As seen in Table 4, there were no statistically significant results regarding recent weight change and the presence of osteoporosis or osteopenia. However, during the early stage, staying an obesity-stable body weight had a protective effect on osteoporosis (OR = 0.26, 95% CI 0.08–0.77, *P* = 0.016) and osteopenia (OR = 0.46, 95% CI 0.25–0.84, *P* = 0.011). We also observed a beneficial association between overweight-stable and osteopenia (OR = 0.53, 95% CI 0.34–0.83, *P* = 0.005). Moreover, increasing weight during the early stage was also found to decrease the likelihood of developing osteoporosis by approximately 30% (OR = 0.71, 95% CI 0.51–0.97, *P* = 0.033).

**Table 4:** The association between weight change in different life periods and osteoporosis/osteopenia

Normal-stable	Osteoporosis						Osteopenia					
	Model 1	*P*-value	Model 2	*P*-value	Model 3	*P*-value	Model 1	*P*-value	Model 2	*P*-value	Model 3	*P*-value
	Ref.		Ref.		Ref.		Ref.		Ref.		Ref.	
Early-stage weight change												
Overweight-stable	0.45 (0.22,0.92)	**0.029**	0.47 (0.22,0.98)	**0.044**	0.48 (0.23,1.01)	0.055	0.52 (0.34,0.80)	**0.002**	0.53 (0.34,0.81)	**0.004**	0.53 (0.34,0.83)	**0.005**
Obesity-stable	0.32 (0.12,0.84)	**0.021**	0.25 (0.08,0.74)	**0.012**	0.26 (0.08,0.77)	**0.016**	0.46 (0.27,0.79)	**0.005**	0.44 (0.24,0.80)	**0.007**	0.46 (0.25,0.84)	**0.011**
Decrease	0.81 (0.49,1.33)	0.100	0.73 (0.44,1.21)	0.220	0.78 (0.47,1.29)	0.327	0.84 (0.56,1.26)	0.399	0.85 (0.56,1.29)	0.446	0.92 (0.60,1.40)	0.690
Increase	0.65 (0.48,0.89)	**0.007**	0.70 (0.51,0.96)	**0.026**	0.71 (0.51,0.97)	**0.033**	0.77 (0.59,1.01)	0.058	0.78 (0.58,1.04)	0.093	0.80 (0.59,1.07)	0.134
Recent weight change												
Overweight-stable	0.79 (0.51,1.22)	0.287	0.77 (0.49,1.22)	0.270	0.76 (0.48,1.20)	0.242	0.87 (0.68,1.12)	0.284	0.89 (0.69,1.13)	0.332	0.88 (0.69,1.13)	0.323
Obesity-stable	1.49 (0.72,3.09)	0.286	1.35 (0.63,2.89)	0.433	1.33 (0.61,2.90)	0.479	0.91 (0.67,1.24)	0.543	0.92 (0.67,1.27)	0.618	0.96 (0.69,1.33)	0.802
Decrease	0.94 (0.67,1.31)	0.709	0.79 (0.55,1.13)	0.195	0.85 (0.59,1.22)	0.377	0.89 (0.72,1.11)	0.317	0.89 (0.71,1.12)	0.318	0.94 (0.74,1.19)	0.617
Increase	1.13 (0.78,1.64)	0.511	1.03 (0.71,1.49)	0.880	0.97 (0.66,1.43)	0.892	0.98 (0.79,1.21)	0.831	0.99 (0.79,1.24)	0.922	0.99 (0.78,1.25)	0.907

Model 1: adjusted for age, sex, ethnicity, and baseline BMI

Model 2: model 1 plus education, smoking, alcohol drinking, physical activity, and sleep status

Model 3: model 2 plus cancer, diabetes, and total cholesterol

Early-stage: from 25 years old to 10 years ago; recent: from 10 years ago to baseline

*P*-values < 0.05 were indicated in bold

The trajectory graph (Fig. 2) clearly showed that individuals in the rapid increase trajectory had substantial weight gains, especially after the age of 45. Therefore, we further separated these individuals and also named the “rapid increase” in the analysis of recent weight change (Additional file 1: Table S4). Compared to the normal-stable group, those in the rapid increase group had higher odds of osteoporosis (OR = 2.34, 95% CI 1.12–4.92, *P* = 0.024).

### Sensitivity analysis

After excluding the participants with BMI of < 15 and > 50 kg/m^2^, the multinomial logistic regression results remained the same (Additional file 1: Table S5). The E-value for the point estimate and the lower confidence interval were 3.93 and 1.67. Following the suggestion from VanderWeele and Ding [33], the large E-value means that only the unmeasured confounders have a strong association with both the BMI trajectories and incident osteoporosis could the observed OR of 2.25 be explained away, but weak confounders could not do so.

## Discussion

This study examined the association between four BMI trajectories and bone health among individuals aged 50 and above in NHANES 2005–2018. We found that a rapid increase in BMI trajectory was moderately and positively associated with osteoporosis and osteopenia. This association was consistent across different sexes and the age group of ≤ 65 years. Additionally, we found that the impact of rapid weight gain on bone loss varied depending on the skeletal site, with the lumbar spine being more affected. When examining the impact of different life-stage weight changes on bone loss, we discovered that maintaining a stable obesity or overweight BMI during early stages was associated with lower odds of osteoporosis and osteopenia compared to those with a normal-stable BMI.

Despite the positive correlation between BMI and BMD, an increasing number of research have revealed that obesity may not be a protective factor against osteoporosis and may even be harmful to bone health [34, 35]. The inconsistent results may be due to the interaction between body weight, lean mass, and fat mass, as well as the complicated relationship between obesity and bone. This includes the positive impact of increased mechanical loading on BMD and the negative impact of excess fat on bone metabolism [8]. Therefore, the relationship between obesity and bone health is not straightforward and requires further investigation. Our study results indicate that individuals who experienced a rapid and extensive increase in BMI trajectory during adulthood may have a higher risk of developing osteoporosis. This finding is consistent with a previous study that found extreme obesity to be associated with reduced BMD in postmenopausal women [36]. Influenced by age-related declines in physical activity and hormone levels, weight gain in middle-aged and elderly people is primarily manifested as an increase in adipose tissue rather than lean tissue [37]. However, excessive adipose tissue is hazardous for bones. A population-based cohort study of Australians aged 45 to 70 found that visceral fat mass was adversely related to BMD after adjusting for body mass and lifestyle factors [38]. This negative association between fat mass and BMD was also confirmed by Zhang et al. [39]. Excess fat mass leads to an imbalance between the pro-inflammatory and anti-inflammatory adipokines, rendering the body in a state of chronic inflammation [40]. High levels of inflammatory factors such as tumor necrosis factor α (TNF-α) and interleukin-6 (IL-6) in fat tissue can promote the differentiation and activation of osteoclasts, inhibit the activity of osteoblasts, and finally lead to the decrease in bone density [41].

Different from the individuals who had rapid and extensive weight gain, those who maintained a stable weight in the early stage, whether overweight or obese, showed a lower risk of osteoporosis or osteopenia. Regardless of the reasons mentioned above, biological homeostasis may play a role. Homeostasis refers to the body’s ability to maintain relative stability in the internal environment despite external stimuli [42]. As individuals age, weight gain, muscle loss, and body fat redistribution can cause an imbalance in homeostasis, ultimately leading to adverse health outcomes. One study found that women who maintain a stable body weight can reduce bone loss after menopause [43]. In our analysis of the early-stage weight change, we found the increase group also had a lower risk of osteoporosis. This may be because most people in this group followed moderate weight gain and had a higher BMD.

Li’s research revealed an inverted U-shaped relationship between BMI and lumbar BMD, indicating that excessive BMI may have negative effects on the lumbar spine [6]. Our study reported that rapid and excessive weight increase affected the lumbar spine, but not the femoral neck. Previous pharmacological investigations have shown that the spine is more responsive to drug therapy than the hip [44]. Similarly, the lumbar spine may be more sensitively influenced by weight change or fat tissue than the femoral neck, since the spine bone has a higher turnover rate and is more sensitive to hormone and endocrine changes [45]. Additionally, a 14-year longitudinal study concluded that body mass index was adversely correlated with bone loss in the lumbar spine, but not in the femoral neck [46]. Yang’s prospective cohort study also found a significant association between whole body fat mass and bone loss only in the lumbar spine [47].

To our knowledge, this is the first study to assess the association between BMI trajectories and osteoporosis and osteopenia. The use of BMI at multiple time points to identify weight change patterns provided further and novel insight into the study of the BMI's impacts on bone. However, our study has some limitations. First, four trajectories of BMI change were identified in this study, but other trajectories may exist. Further identification of potential patterns of BMI change is needed. Second, the small number of people in the increase to decrease group may lead to increased instability of the results. Third, in the cross-sectional study of NHANES study, causality could not be established. Follow-up cohort studies or randomized clinical trials are needed for causal research. Meanwhile, weight history was collected retrospectively so that recall bias may exist. Last, we conducted this research in the American population, further validation in other populations is needed.

## Conclusion

The observational study from NHANES 2005–2018 showed that rapid and excess weight gain throughout adulthood may increase the risk of osteoporosis. The lumbar spine BMD is more responsive to quick and excessive weight increase than the femoral neck. Maintaining an early-stage stable and overweight BMI is a protective factor against osteoporosis. This study suggests that individuals should avoid fast weight increases and extreme obesity to promote bone health and prevent osteoporosis.

### Supplementary Information

**Additional file 1: Table S1.** Model adequacy assessments of latent class trajectory models for the total population. **Table S2.** Model adequacy assessments of latent class trajectory models for male. **Table S3.** Model adequacy assessments of latent class trajectory models for female. **Table S4.** The association between recent weight change and osteoporosis/osteopenia. **Table S5.** The association between BMI trajectories and osteoporosis/osteopenia after excluding participants with BMI < 15 and > 50 kg/m^2^. **Figure S1.** BMI trajectories during adulthood of different sexes. Figure A for male and B for female.

## Data Availability

The NHANES database is publicly accessible and contains deidentified data for all participants utilized in this study. This dataset can be accessed, queried, and downloaded using this link: https://www.cdc.gov/nchs/nhanes/index.htm.

## Abbreviations

BMI: Body mass index
BMD: Bone mineral density
NHANES: National Health and Nutrition Examination Survey
OR: Odds ratio
CI: Confidence interval
LCTM: Latent class trajectory model
NCHS: The National Center for Health Statistics
IQR: Interquartile range
DXA: Dual-energy X-ray Absorptiometry
WHO: World Health Organization
ISCD: The International Society for Clinical Densitometry
CDC: Centers for disease control and prevention
MET: Metabolic equivalents of task
BP: Blood pressure
SE: Standard error
ANOVA: Analysis of variance
DBP: Diastolic blood pressure
TNF-α: Tumor necrosis factor α
IL-6: Interleukin-6
