# Supplementary material for: BMI trajectory of rapid and excessive weight gain during adulthood is associated with bone loss: a cross-sectional study from NHANES 2005–2018
Source: J Transl Med. 2023 Aug 12;21:536. doi: 10.1186/s12967-023-04397-9 (PMC10422827; doi:10.1186/s12967-023-04397-9)
Supplement: Supplementary file 1 — Additional file 1: Table S1. Model adequacy assessments of latent class trajectory models for the total population. Table S2. Model adequacy assessments of latent class trajectory models for male. Table S3. Model adequacy assessments of latent class trajectory models for female. Table S4. The association between recent weight change and osteoporosis/osteopenia. Table S5. The association between BMI trajectories and osteoporosis/osteopenia after excluding participants with BMI < 15 and > 50 kg/m2. Figure S1. BMI trajectories during adulthood of different sexes. Figure A for male and B for female. [file 12967_2023_4397_MOESM1_ESM.docx]

**BMI Trajectory of Rapid and Excessive Weight Gain during Adulthood is Associated with Bone Loss— A Cross-sectional Study from NHANES 2005-2018**

Jiacheng Wang^1^, Yi Zheng ^2^, Yawen Wang ^1^, Chengjun Zhang ^1^, Yanfeng Jiang^2,3^, Chen Suo ^1,3^, Mei Cui^5^, Tiejun Zhang ^1,3,6^, Xingdong Chen ^2,3,4,6*^, and Kelin Xu^1,3*^

**Affiliation:**

1 School of Public Health, and the Key Laboratory of Public Health Safety of Ministry of Education, Fudan University, Shanghai, China;

2 State Key Laboratory of Genetic Engineering, Human Phenome Institute, Fudan University, Shanghai, China;

3 Fudan University Taizhou Institute of Health Sciences, Taizhou, Jiangsu, China;

4 National Clinical Research Center for Aging and Medicine, Huashan Hospital, Fudan University, Shanghai, China;

5 Department of Neurology, Huashan Hospital, Fudan University, Shanghai, China;

6 Yiwu Research Institute of Fudan University, Yiwu, Zhejiang, China.

**Corresponding Author:** Kelin Xu, Ph.D., School of Public Health, and the Key Laboratory of Public Health Safety of Ministry of Education, Fudan University, Shanghai 200000, China ([xukelin@fudan.edu.cn](mailto:xukelin@fudan.edu.cn), Tel: +86-177-1739-3652); Xingdong Chen, Ph.D., State Key Laboratory of Genetic Engineering, Human Phenome Institute, Fudan University, Shanghai 200000, China ([xingdongchen@fudan.edu.cn](mailto:xingdongchen@fudan.edu.cn), Tel: +86-135-6479-3419).

**Additional file 1: Table S1 Model adequacy assessments of latent class trajectory models for the total population**

|  |  | Mean posterior probability | | | | | | |  | Class size of the population (%) | | | | | | |
| --- | --- | --- | --- | --- | --- | --- | --- | --- | --- | --- | --- | --- | --- | --- | --- | --- |
| Class | BIC | class1 | class2 | class3 | class4 | class5 | class6 | class7 |  | class1 | class2 | class3 | class4 | class5 | class6 | class7 |
| 1 | 207912.49 | 1 |  |  |  |  |  |  |  | 100 |  |  |  |  |  |  |
| 2 | 205684.4 | 0.98 | 0.87 |  |  |  |  |  |  | 93.93 | 6.07 |  |  |  |  |  |
| 3 | 203534.81 | 0.96 | 0.83 | 0.87 |  |  |  |  |  | 87.67 | 6.11 | 6.22 |  |  |  |  |
| 4 | 202578.08 | 0.78 | 0.91 | 0.87 | 0.82 |  |  |  |  | 12.91 | 79.14 | 2.01 | 5.94 |  |  |  |
| 5 | 202028.11 | 0.90 | 0.75 | 0.80 | 0.88 | 0.81 |  |  |  | 4.45 | 25.95 | 62.62 | 1.25 | 5.73 |  |  |
| 6 | 201716.07 | 0.79 | 0.77 | 0.75 | 0.75 | 0.88 | 0.81 |  |  | 3.98 | 5.46 | 47.21 | 35.96 | 1.37 | 6.03 |  |
| 7 | 201407.48 | 0.79 | 0.77 | 0.72 | 0.75 | 0.72 | 0.88 | 0.85 |  | 4.29 | 5.55 | 7.06 | 37.90 | 42.53 | 1.40 | 1.27 |

|  |  | Mean posterior probability | | | | | | |  | Class size of the population (%) | | | | | | |
| --- | --- | --- | --- | --- | --- | --- | --- | --- | --- | --- | --- | --- | --- | --- | --- | --- |
| Class | BIC | class1 | class2 | class3 | class4 | class5 | class6 | class7 |  | class1 | class2 | class3 | class4 | class5 | class6 | class7 |
| 1 | 112553.80 | 1.00 |  |  |  |  |  |  |  | 100.00 |  |  |  |  |  |  |
| 2 | 111501.71 | 0.88 | 0.82 |  |  |  |  |  |  | 83.36 | 16.64 |  |  |  |  |  |
| 3 | 110341.25 | 0.86 | 0.97 | 0.86 |  |  |  |  |  | 4.67 | 89.81 | 5.51 |  |  |  |  |
| 4 | 109958.26 | 0.84 | 0.75 | 0.83 | 0.85 |  |  |  |  | 4.09 | 20.40 | 71.77 | 3.74 |  |  |  |
| 5 | 109699.76 | 0.77 | 0.75 | 0.78 | 0.83 | 0.88 |  |  |  | 4.40 | 26.21 | 63.84 | 4.46 | 1.10 |  |  |
| 6 | 109594.24 | 0.76 | 0.72 | 0.69 | 0.75 | 0.89 | 0.83 |  |  | 4.18 | 8.56 | 43.92 | 37.89 | 1.02 | 4.42 |  |
| 7 | 109578.12 | 0.73 | 0.76 | 0.70 | 0.54 | 0.71 | 0.88 | 0.81 |  | 4.35 | 4.15 | 32.36 | 31.37 | 22.06 | 1.00 | 4.71 |

**Additional file 1: Table S2 Model adequacy assessments of latent class trajectory models for male**

|  |  | Mean posterior probability | | | | | | |  | Class size of the population (%) | | | | | | |
| --- | --- | --- | --- | --- | --- | --- | --- | --- | --- | --- | --- | --- | --- | --- | --- | --- |
| Class | BIC | class1 | class2 | class3 | class4 | class5 | class6 | class7 |  | class1 | class2 | class3 | class4 | class5 | class6 | class7 |
| 1 | 93392.00 | 1.00 |  |  |  |  |  |  |  | 100.00 |  |  |  |  |  |  |
| 2 | 92269.74 | 0.97 | 0.88 |  |  |  |  |  |  | 89.57 | 10.43 |  |  |  |  |  |
| 3 | 91354.30 | 0.81 | 0.94 | 0.87 |  |  |  |  |  | 8.61 | 81.75 | 9.65 |  |  |  |  |
| 4 | 90794.26 | 0.81 | 0.81 | 0.92 | 0.91 |  |  |  |  | 8.06 | 14.96 | 74.75 | 2.22 |  |  |  |
| 5 | 90503.37 | 0.90 | 0.78 | 0.93 | 0.82 | 0.82 |  |  |  | 1.82 | 7.21 | 77.78 | 6.22 | 6.97 |  |  |
| 6 | 90332.85 | 0.79 | 0.81 | 0.75 | 0.76 | 0.89 | 0.81 |  |  | 5.22 | 5.91 | 38.30 | 42.67 | 1.65 | 6.24 |  |
| 7 | 90219.45 | 0.79 | 0.82 | 0.66 | 0.66 | 0.81 | 0.90 | 0.82 |  | 4.61 | 5.89 | 9.41 | 20.31 | 55.72 | 1.58 | 2.48 |

**Additional file 1: Table S3 Model adequacy assessments of latent class trajectory models for female**

|  | Osteoporosis | | | | | | | |  | Osteopenia | | | | | | | |  |
| --- | --- | --- | --- | --- | --- | --- | --- | --- | --- | --- | --- | --- | --- | --- | --- | --- | --- | --- |
|  | Model 1 | *P*-value | Model 2 | *P*-value | Model 3 | *P*-value | Model 4 | *P*-value |  | Model 1 | *P*-value | Model 2 | *P*-value | Model 3 | *P*-value | Model 4 | *P*-value | |
| Normal-stable | ref |  | ref |  | ref |  | ref |  |  | ref |  | ref |  | ref |  | ref |  | |
| Overweight-stable | 0.84(0.55,1.29) | 0.421 | 0.82(0.52,1.28) | 0.378 | 0.80(0.51,1.27) | 0.352 | 0.84(0.52,1.33) | 0.454 |  | 0.89(0.70,1.14) | 0.362 | 0.91(0.71,1.15) | 0.421 | 0.91(0.71,1.16) | 0.426 | 0.95(0.74,1.21) | 0.654 | |
| Obesity-stable | 1.71(0.81,3.58) | 0.156 | 1.54(0.72,3.30) | 0.270 | 1.53(0.69,3.37) | 0.297 | 1.63(0.72,3.70) | 0.239 |  | 0.96(0.71,1.30) | 0.795 | 0.98(0.72,1.34) | 0.889 | 1.03(0.75,1.41) | 0.865 | 1.09(0.80,1.49) | 0.568 | |
| Decrease | 0.96(0.69,1.34) | 0.826 | 0.81(0.57,1.15) | 0.245 | 0.87(0.60,1.26) | 0.465 | 0.91(0.63,1.31) | 0.602 |  | 0.91(0.73,1.13) | 0.395 | 0.91(0.72,1.14) | 0.396 | 0.96(0.76,1.22) | 0.755 | 1.00(0.78,1.28) | 0.990 | |
| Increase | 1.15(0.80,1.67) | 0.455 | 1.04(0.72,1.51) | 0.821 | 0.99(0.67,1.46) | 0.959 | 0.97(0.66,1.43) | 0.879 |  | 0.99(0.79,1.23) | 0.908 | 1.00(0.80,1.25) | 0.994 | 1.00(0.79,1.27) | 0.999 | 0.99(0.78,1.25) | 0.924 | |
| Rapid increase | 2.90(1.47,5.72) | **0.002** | 2.47(1.25,4.89) | **0.009** | 2.48(1.22,5.04) | **0.012** | 2.34(1.12,4.92) | **0.024** |  | 1.30(0.87,1.94) | 0.204 | 1.35(0.89,2.03) | 0.158 | 1.41(0.92,2.15) | 0.117 | 1.32(0.86,2.02) | 0.202 | |

**Additional file 1: Table S4 The association between recent weight change and osteoporosis/osteopenia**

Model 1: age, sex, ethnicity and baseline BMI.

Model 2: model 1 plus education, smoking, alcohol drinking, physical activity and sleep status.

Model 3: model 2 plus cancer, diabetes and total cholesterol.

Model 4: model 3 plus BMI at 25 years

*P*-value <0.05 were indicated in bold.

**Additional file 1: Table S5 The association between BMI trajectories and osteoporosis/osteopenia after excluding participants with BMI <15 and >50 Kg/m^2^**

|  | Osteoporosis |  |  |  |  |  |  | Osteopenia |  |  |  |  |  |
| --- | --- | --- | --- | --- | --- | --- | --- | --- | --- | --- | --- | --- | --- |
|  | model1 | *P*-value | model2 | *P*-value | model3 | *P*-value |  | model1 | *P*-value | model2 | *P*-value | model3 | *P*-value |
| Stable |  |  | ref |  | ref |  |  | ref |  | ref |  | ref |  |
| Slight increase | 0.94(0.66,1.33) | 0.720 | 0.82(0.58,1.17) | 0.279 | 0.94(0.65,1.37) | 0.759 |  | 1.02(0.83,1.25) | 0.879 | 1.01(0.82,1.24) | 0.910 | 1.12(0.89,1.41) | 0.325 |
| Increase to decrease | 0.66(0.28,1.59) | 0.355 | 0.51(0.20,1.30) | 0.161 | 0.65(0.26,1.61) | 0.349 |  | 0.79(0.45,1.36) | 0.392 | 0.78(0.44,1.36) | 0.380 | 0.80(0.51,1.27) | 0.352 |
| Rapid increase | 2.33(1.26,4.29) | **0.007** | 2.09(1.11,3.93) | **0.023** | 2.28(1.20,4.32) | **0.012** |  | 1.34(0.93,1.93) | 0.111 | 1.38(0.95,2.00) | 0.095 | 1.48(1.01,2.17) | **0.046** |

Model 1: age, sex, ethnicity and baseline BMI.

Model 2: model 1 plus education, smoking, alcohol drinking, physical activity and sleep status.

Model 3: model 2 plus cancer, diabetes and total cholesterol.

*P*-value <0.05 were indicated in bold.

A

B

**Additional file 1: Figure S1 BMI trajectories during adulthood of different sexes. Figure A for male and B for female.**

The solid lines are the predicted BMI trajectory, and the dashed lines are the 95% confidence interval; BMI: body mass index.
